# Lung function trajectories in children with early diagnosis of non-cystic fibrosis bronchiectasis: a retrospective observational study

**DOI:** 10.1186/s13052-024-01799-3

**Published:** 2024-11-14

**Authors:** Rossella Lamberti, Simona Ferraro, Andrea Farolfi, Michele Ghezzi, Salvatore Zirpoli, Alice Marianna Munari, Sai Spandana Adivishnu, Giuseppe Marano, Elia Biganzoli, Gian Vincenzo Zuccotti, Enza D’Auria

**Affiliations:** 1 Department of Pediatrics, Buzzi Children’s Hospital, Milan, Italy; 2 Department of Biomedical and Clinical Sciences, Buzzi Children’s Hospital, University of Milan, Milan, Italy; 3 Radiology Department, “Vittore Buzzi” Children’s Hospital, Milan, Italy

**Keywords:** Children, Non-cystic fibrosis bronchiectasis, Lung function

## Abstract

**Background:**

Non-cystic fibrosis (non-CF) bronchiectasis (BE) is defined as a clinical syndrome of recurrent, persistent wet cough and abnormal bronchial dilatation on chest High Resolution Computed Tomography (HRCT) scans. The aims of this study were to characterize the pattern of the trajectories of lung function parameters and to consider the relationship between the lung function and radiological severity according to the modified Reiff score.

**Methods:**

The study retrospectively considered 86 children (46.5% male, median age of 4 years) with non-CF BE, admitted at the Paediatric Pneumology Unit of Buzzi Children’s Hospital from January 2015 to December 2022. The diagnosis of BE was made according to the presence of a suggestive clinical history and symptoms and key features of BE evidenced on chest HRCT scans. The modified Reiff score was adapted to quantify the severity of BE. Spirometry (*COSMED MicroQuark* spirometer) was performed at median age of 5.78 years (baseline or T_0_) and after 1 and 2 years from the baseline (T_1_ and T_2,_ respectively). The general trends of lung function parameters were estimated by ANOVA models for repeated measurements. For each lung function parameter, a longitudinal regression model was fitted. The analysis was performed with the software R release 4.2.3. The statistical significance was deemed when the p-value resulted lower than 0.05.

**Results:**

The general trends of lung function parameters showed a statistically significant variation of forced vital capacity (FVC%) and forced expiratory volume in 1s (FEV_1_%) from T_0_ to T_1_ (*p* = 0.0062, 0.0009) and no significant change for FVC%, FEV_1_% and forced expiratory flow 25–75% of VC (FEF_25/75_%) from T_1_ to T_2_ (*p* = 0.145, 0.210, 0.600, respectively). Notably, we found no correlation between the age at diagnosis and the lung function parameters at T_0_ (*r* = 0.149, 0.103 and 0.042 for FVC%, FEV_1_% and FEF_25/75_%, respectively). Instead, a poor negative correlation resulted between the Reiff score and FVC%, FEV_1_% e FEF_25/75_% at baseline (Spearman coefficients: rho=-0.156, -0.204, -0.103, respectively).

**Conclusions:**

A stable pulmonary function is detectable within 2 years follow up from baseline spirometry. The modified Reiff score should be considered as a good tool not only to quantify the radiological lung involvement but also the degree of pulmonary function impairment.

## Introduction

Non-cystic fibrosis (non-CF) bronchiectasis (BE) is defined as a clinical syndrome of recurrent, persistent wet or productive cough, airway infection and inflammation and abnormal bronchial dilatation on chest High Resolution Computed Tomography (HRCT) scans [1].

Published data suggest that non-CF BE prevalence ranges widely (0.2–735 cases per 100000 children) across different geographical areas, and this has been associated to various causes, including large disparities in the standards of care, the high risk on neglecting the disease and the different air pollution exposure [1, 2].

More than 3 episodes per year of protracted bacterial bronchitis and a wet or productive cough failing to respond to 4 weeks of oral antibiotics may predict the presence of abnormal bronchial dilatation (adjusted odds ratio [OR] of 11.5, (95% CI 2.3–56.0) and of 20.9, (95% CI 5.4–81.8), respectively [3, 4].

Neglected or poorly managed BE in childhood may result in progressive lung function decline, and abnormal spirometry results in childhood are associated with significant respiratory and cardiovascular morbidity and mortality at older ages [5–7]. More than 60% of adults with BE have been reported to have symptoms from childhood [7].

The proportion of resolution or improvement may be as great as 64% although it may vary with BE severity, underlying etiology, treatment provided and the diagnostic criteria used [8, 9].

The main objectives of managing children with bronchiectasis are: optimize lung growth and preserve lung function, optimize Quality of Life, minimize exacerbations, prevent complications and if possible, reverse structural lung injury [1, 10, 11].

There is evidence that in some children BE is reversible and/or preventable, and early identification and management are crucial factors for the resolution or improvement rates [1, 3, 12–14].

To assess the impact of early diagnosis and management of BE in children, longitudinal studies should characterize the pattern of lung function trajectory during disease monitoring [10, 15, 16].

Current evidence available on serial lung function measurements in childhood are few and heterogeneous [15, 17–20].

From such contrasting results [15, 17–20], it emerges the need to define a tailored management of BE to preserve lung function, identifying the predictive variables related to clinical improvement.

The first aim of the present study was to characterize the pattern of the trajectories of lung function parameters in a population of young children with non-CF BE.

The second aim was to consider the relationship of lung function parameters with the radiological severity of BE at diagnosis, according to the modified Reiff score [21–24].

## Study design

This retrospective cohort study considered children with non-CF BE admitted at the Paediatric Pneumology Unit of Buzzi Children’s Hospital from January 2015 to December 2022.

We retrieved from medical records demographic, clinical and instrumental data including: auxological parameters (Body Mass Index, BMI z-score), respiratory parameters (forced vital capacity - FVC%, forced expiratory volume in 1s - FEV_1_%, forced expiratory flow 25–75% of VC - FEF_25/75_%) and radiological evaluation of severity of BE according to the modified Reiff score. Moreover, atopy diathesis, number of respiratory exacerbations with and without hospitalization during follow-up and systemic antibiotics prophylaxis, inhaler bronchodilator or corticosteroids prescription were considered.

### Inclusion criteria

- suggestive clinical history, symptoms and key features of BE on HRCT scans;
- availability of respiratory parameters (FVC%, FEV_1_% and FEF_25/75_%) at scheduled intervals (i.e. baseline - T_0_ and after 1 and 2 years from baseline - T_1_ and T_2,_ respectively) in clinical well-being.

### Exclusion criteria

- diagnosis of BE based on Magnetic Resonance Imaging (MRI);
- unavailability of respiratory parameters in children younger than 5 years old unable to correctly perform the spirometry;
- other confirmed diagnoses (i.e. CF or Primary Ciliary Dyskinesia - PCD).

Data were retrospectively evaluated according to the principles of the Declaration of Helsinki as revised in 2008. Ethical committee approval was not requested because the General Authorization to Process Personal Data for Scientific Research Purposes (Authorization no. 9/2014) declared that ethics approval is not needed for retrospective archive studies that use ID codes, preventing the data from being traced back directly to the data subject. The privacy of the collected information was ensured according to Regulation (EU)/2016/679 GDPR (Regulation (EU) 2016/679), Legislative Decree n.101/18.

## Methods

This retrospective cohort study aimed to evaluate lung function following a diagnosis of non-CF BE in children admitted at the Paediatric Pneumology Unit of Buzzi Children’s Hospital from January 2015 to December 2022.

According to recent international guidelines [1, 12], the diagnosis of BE was made according to the presence of a suggestive clinical history and symptoms and key features of BE evidenced on HRCT [1, 12]. HRCT as gold standard for the diagnosis of BE, allowed to define its exact localization and distribution, thus addressing its etiology [1, 12, 25–27]. For the diagnosis of bronchiectasis, we used broncho-arterial ratio (BAR, inner airway compared to outer vessel diameter), with a cut-off of ≥ 0.8 used to define abnormality [12].

We excluded patients with BE diagnosed by chest MRI and not confirmed by HRCT to ensure data consistency [1, 12, 25–27]. In fact, including cases based only on MRI could introduce diagnostic variability with potential false positives or non-comparable diagnoses [28]. HRCT images were evaluated separately by two pediatric radiologists with more than 5 years of clinical experience, by using the modified Reiff score to identify the severity of BE [11].

In the few instances of disagreement between the radiologists, a consensus was reached through discussion to resolve the discordance. Therefore, no formal statistical analysis of inter-rater agreement was conducted.

The modified Reiff score (range 0–18), used to quantify the severity of BE, was based on the number of lobes involved (six lung lobes, including lingula as a separate lobe) and on the severity of bronchial dilatation (0 = none; 1 = tubular, 0.8 ≤ BAR < 2; 2 = varicose, 2 ≤ BAR < 3; 3 = cystic, BAR ≥ 3), with a maximum score of 18. This score was subdivided into mild [1–6], moderate [7–12], and severe [21–24].

The selected patients underwent laboratory tests and instrumental investigations for a correct differential diagnosis.

The diagnosis of CF was excluded by negative sweat test and/or genetic analysis.

PCD was excluded by nasal cytology and/or genetic analysis by the electronic microscopic evaluation of nasal cilia biopsy and/or immunofluorescence staining and/or genetic analysis and/or decreased nasal nitric oxide level measurement and PICADAR score > 5. Nasal nitric oxide cutoff value for PCD was defined at 77 nl/minute [29]. 

The post-infectious etiology was considered on the basis of the clinical history and the identification of infectious agents through culture examination of the bronchoalveolar lavage fluid (data available for 33 patients out of 86 who underwent bronchoscopy). Measurement of plasma immunoglobulins (IgA, IgM, IgG) and study of lymphocyte subpopulations were performed to exclude immunological deficits.

Furthermore, Mantoux intradermal testing, quantiferon and plasma alpha 1-antitrypsin measurement were also performed. For patients with gastroesophageal reflux symptoms, ph-impedance measurement was performed to confirm the diagnosis.

The diagnosis of malformation - including congenital heart disease and esophageal atresia - was suspected in the prenatal period and confirmed at birth through appropriate diagnostic investigations.

The auxological and pulmonary function parameters of all patients were further retrieved considered.

BMI z-score was estimated by using the *World Health Organization* (WHO) classification. According to the BMI z-score reference values, all subjects were classified into normal weight (NW, -2 ≤ BMI z-score ≤ 1) and overweight/obese (BMI z-score > 1 and ≥ 2, respectively) [30].

Spirometry (COSMED MicroQuark spirometer) was performed according to the criteria of the *American Thoracic Society* (ATS) and the *European Respiratory Society* (ERS) by trained pediatric nurses at scheduled intervals (i.e. T_0_, T_1_ and T_2_) [31, 32]. The spirometry was performed in clinical well-being. The measurements included FVC%, FEV_1_% and FEF_25/75_% (as percentage of the predicted normal values).

Asthma diagnosis was made in patients with history of wheezing, shortness of breath, chest tightness, and/or by variable expiratory airflow limitation, confirmed by reversibility test (FEV_1_% after bronchodilation ≥ 12%) according to *Global Initiative for Asthma* (GINA) indications [33].

The atopic state was detected via percutaneous and/or IgE tests specific to common seasonal and perennial inhalants.

Respiratory exacerbation was defined by any of the following: change in cough quality from dry to wet and/or sputum production for ≥ 3 days, breathlessness, chest pain, crepitations, wheezing with or without an increase in values of infectious markers [1].

Once non-CF BE was confirmed, all patients began daily sessions of respiratory physiotherapy personalized based on age and individual abilities [1, 34, 35].

In case of respiratory exacerbation (increased cough and/or increased quantity of sputum and/or purulence for more than 3 days) [1], systemic antibiotic treatment was prescribed for a duration of 10–14 days based on available microbial data or empirically (amoxicillin-clavulanate, cefpodoxime).

Due to the youngest age and the invasivity of the procedure, bronchoscopy and bronchoalveolar lavage analysis (microbiology) was performed only in a minority of cases (38.4% of patients, resulting negative in 84% of the tests). Due to the study design, no data are available about blood culture and viral panel test from medical records considered.

Hospitalization was made in patients with compromised general conditions, need for oxygen therapy and intravenous antibiotic therapy (ampicillin-sulbactam, ceftriaxone, cefotaxime).

According to ERS guidelines [1, 36–39], long-term antibiotic treatment (azithromycin 3 times a week for a minimum of 6 months) was prescribed to patients with more than one hospital admission or more than 3 respiratory exacerbations in the previous 12 months.

Inhaled steroids with or without long-acting beta-agonist bronchodilators were administered in patients with a positive broncho-reversibility test and personalized respiratory physiotherapy sessions were intensified during an exacerbation [1].

### Statistical analysis

Categorical variables were reported using counts and percentages. Numerical variables were reported using either mean and standard deviation or median and quartiles, depending on the empirical distribution showing a unimodal and symmetrical shape or not. The correlation of age at presentation (T_0_) and the modified Reiff score with lung parameters at the same time was evaluated by scatterplots and by the Pearson correlation coefficient r. The association of further hospitalization, coded as categorical variable with two values (0 = no, 1 = yes), with the lung parameters above was evaluated by boxplots.

To assess the changes in time of the respiratory parameters, methods for longitudinal data analysis with a categorical time variable were used [40]. The time variable consisted of three distinct occasions: T_0_ (baseline), T_1_ and T_2_ after 1 and 2 years from baseline). First, the general trends of lung function parameters (FVC%, FEV_1_% and FEF_25/75_%) were estimated by ANOVA (Analysis of Variance) models for repeated measurements. Estimates and 95% CIs of the average differences between consecutive occasions were reported; the statistical significance was assessed by the Wald test (z distribution). After this, analysis of response profiles [40] was performed. For each lung function parameter, a longitudinal regression model was fitted, with differences between respiratory parameters (T_1_-T_0_, T_2_-T_0_) as response variable, and time (specified through dummy coding), baseline measurement (i.e. the value of observed at T_0_) and the age of child at presentation (T_0_) as explanatory variables. In particular, the latter one allows to account for the influence of physiological growth on the measurements of lung function parameters of the same child through distinct years. Non-linearity and interaction effects of the above explanatory variables were assessed using the Wald test, and, where non-significant, were removed. Furthermore, the Wald test was also used to assess potential effects of gender, age at diagnosis, and BMI-z on the models.

Concerning the choice of the estimation method for the longitudinal models illustrated above, Generalized Estimating Equation (GEE) methods provide a suitable methodological framework for estimating the trends of lung function parameters at population level [41]. Furthermore, compared to mixed effects modeling methods, GEE has the advantage of being more robust with respect to violations of assumptions about the correlation among repeated measurements. For these reasons, GEE methods were adopted. For each test of hypothesis, the statistical significance was deemed when the p-value resulted lower than 0.05. The analysis was performed with the software R release 4.2.3 [42].

## Results

### Case series description

We firstly retrieved data on 103 patients. Five were excluded for poor compliance with carrying out spirometry (< 5 years of age), and 12 because of a BE diagnosis detected by chest MRI and not by HRCT.

The main features of the selected case series consisting of 86 patients were reported in Table 1.

**Table 1:** Demographic and clinical features of the case series at diagnosis

Age (years): median (25th -75th percentiles)	4 (3–6)
Gender, F, n (%)	46 (53.5%)
N infections/each patient: median (25th -75th percentiles)	4 (3-5.75)
Modified Reiff score: median (25th -75th percentiles)	2 (2–3)
Comorbidities, n (%)	Allergy, 17 (19.7%) Prematurity without BPD, 8 (9.4%) Prematurity with BPD, 6 (6.9%) Congenital Heart defect, 7 (8.3%) Gastroesophageal reflux, 4 (4.8%) Immunodeficiency, 3 (3.4%) Epilepsy, 3 (3.4%) Esophageal atresia, 2 (2.3%)

F: female, n: number, BPD: bronchopulmonary dysplasia

Median age at diagnosis was 4 years (25th -75th percentiles: 3–6), 46.5% were male and 32.5% presented symptoms within 1 year of age. Most of patients had a diagnosis of non-CF BE when were infants or toddlers (30% within 3 years of age). Before BE diagnosis, all patients had at least 2 chest radiographs and the occurrence of 4 lower airway infections.

The main comorbidities were reported in Table 1. In most cases no comorbidities were found; in about 20% of cases, it was reported allergic disease with sensitization to seasonal and perennial allergens detected through percutaneous tests and/or specific IgE tests. Gastro-esophageal reflux disease was recorded in about 5% of patients and about 3% were affected by immunological deficiencies (2 patients with IgA deficiency and one with severe combined immunodeficiency).

**Fig. 1:**
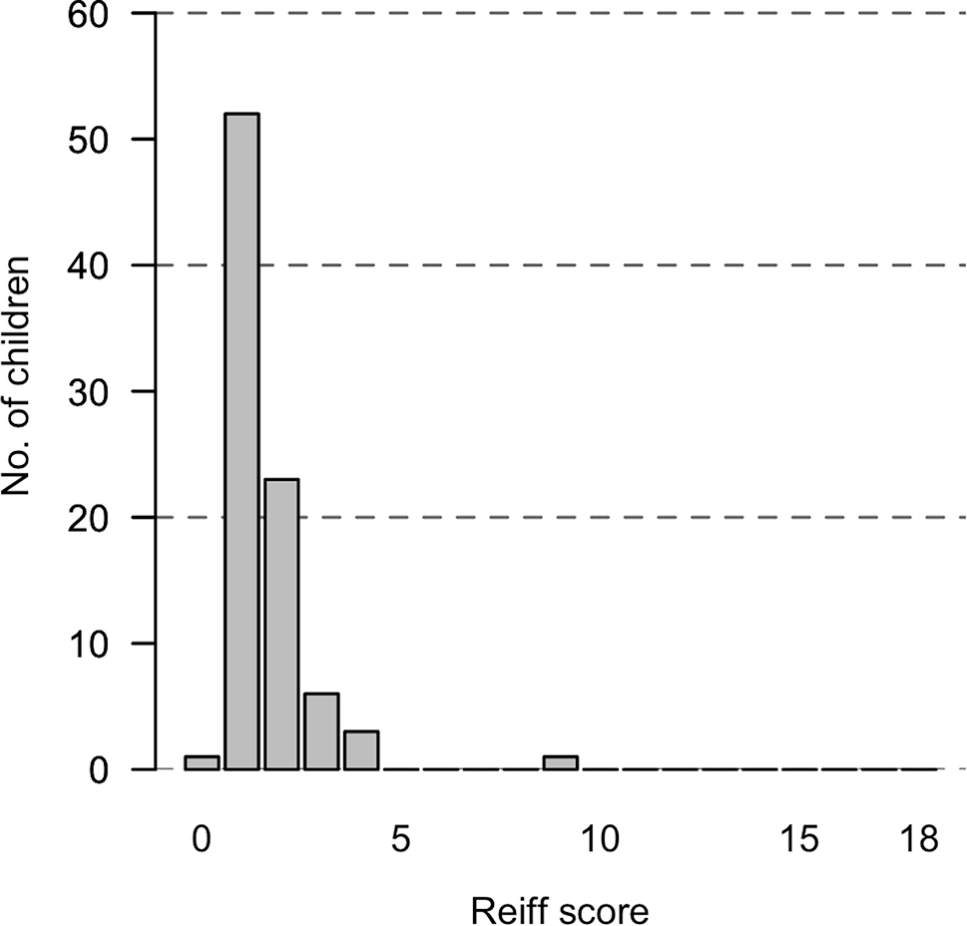
Distribution of Reiff scores across the case series

In Fig. 1 1 we have reported the distribution of the case series according to the modified Reiff score, showing that in this case series the severity of BE may be related to a mild lung impairment.

The first measurement (T_0_) of respiratory parameters was performed at a median age of 5.78 years (5.07–6.73). Repeated spirometry was performed at a median time distance of 1 and 2 years from the baseline (T_1_ and T_2_ respectively), as reported in Table 2.

**Table 2:** Anthropometric and respiratory features of the case series during follow up visits

	BMI z-score	Respiratory parameters °
Baseline T_0_ 5.78 years (5.07–6.73) *	NW, 70 (81.4%) OW, 15 (17.5%) OB, 1 (1.1%)	FVC% 92.05 (13.63) FEV_1_% 91.87 (14.00) FEF_25/75_% 86.60 (16.79)
After 1 year (T_1_) 6.82 (6.07–7.74) *	NW, 71 (82.6%) OW, 14(16.3%) OB, 1 (1.1%)	FVC % 96.67 (13.48) FEV_1_% 95.51 (12.35) FEF_25/75_% 88.1 (15.46)
After 2 years (T_2_) 7.76 (7.07–8.83) *	NW, 70 (81.4%) OW, 14 (16.3%) OB, 2 (2.3%)	FVC % 95.40 (11.70) FEV_1_% 94.17 (11.44) FEF_25/75_% 87.1 (15.87)

NW: normal weight, OW: overweight, OB: obese, FVC %: forced vital capacity, FEV_1_% forced expiratory volume in 1 s, FEF_25/75_% forced expiratory flow 25–75% of vital capacity; *age expressed in years (median), °mean (SD)

At T_0_ most patients (81.4%) were normal weight (-2 ≤ BMI z-score ≤ 1), 17.5% were overweight (1 > BMI z-score < 2) and 1.1% obese (BMI z-score ≥ 2). These percentages did not significantly change after 1 and 2 years from baseline (T_1_ and T_2_ respectively).

By considering location and extent of the disease, middle lobe and left lower lobe were mainly involved (44.1% and 17.4% respectively).

Most patients at T_0_ (69.7%) were treated by three-times-weekly oral azithromycin for six months and after 1 year (T_1_) only 18.6% of children [1, 35–38]. No further treatment at T_2_ was prescribed.

Within 1 year (T_1_) 51% of patients experienced from 1 to 3 recurrences and 2% more than 3 exacerbations. Within 2 years (T_2_) 18.7% had from 1 to 3 recurrences.

### Analysis of lung function parameters longitudinal trends

**Fig. 2:**
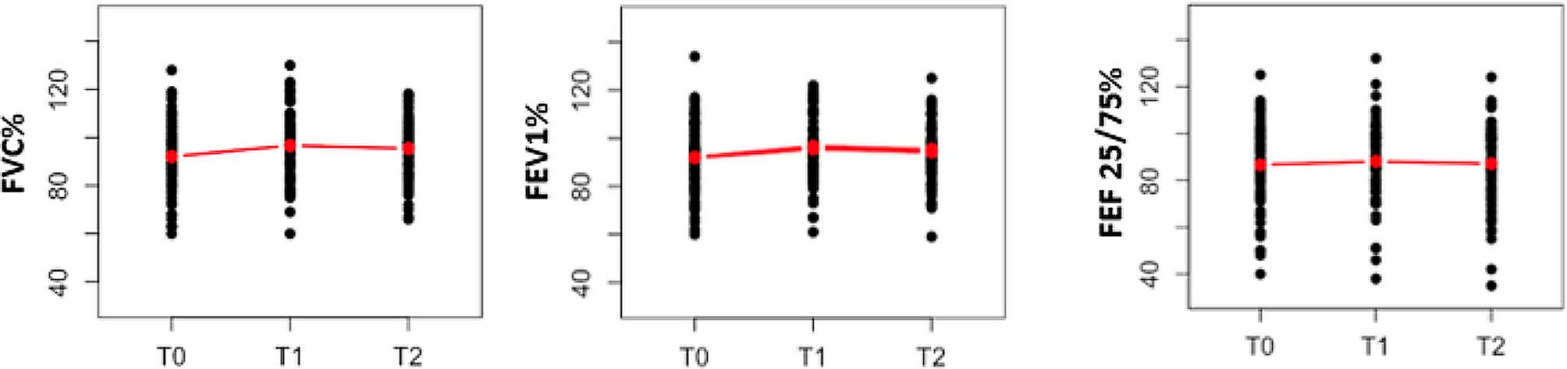
Average trajectories of lung function parameters. Black dots: observed values. Red dots: estimated averages at baseline (T_0_), T_1_ to T_2_. The segments connecting the average values show the general trends

The general trends of lung function parameters showed an increase between T_0_ and T_1_, which was statistically significant only for FVC% and FEV_1_% (*p* = 0.0009, 0.0062), followed by a slight decrease between T_1_ to T_2_, however, not significantly different from T_0_ (*p* = 0.2100, 0.1450, 0.5500, for FVC%, FEV_1_% and FEF_25/75_% respectively). As a consequence, the average lung parameter values at T_2_ results higher than the average at T_0_, although evidence of a statistically significant difference was found for FVC% only (Fig. 2; Table 3).

**Table 3:** Estimates of the coefficients and p values of the changes between serial measurements

		Est, 95% CIs:	*p*
FVC %	**T**_1_ **vs. T**_0_ **T**_**2**_ **vs. T**_1_ **T**_**2**_ **vs. T**_0_	4.63 (1.89, 7.37) -1.28 (-3.28,0.72) 3.35 (0.73, 5.96)	0.0009* 0.210 0.012*
FEV_1_%	**T**_1_ **vs. T**_0_ **T**_**2**_ **vs. T**_1_ **T**_**2**_ **vs. T**_0_	3.64 (1.04, 6.24) -1.34 (-3.14, 0.46) 2.30 (-0.42, 5.02)	0.0062* 0.145 0.0968
FEF_25/75_%	**T**_1_ **vs. T**_0_ **T**_**2**_ **vs. T**_1_ **T**_**2**_ **vs. T**_0_	1.50 (-1. 86, 4.86) -1.00 (-4.26, 2.28) 0.50 (-2.73, 4.76)	0.380 0.550 0.790

Importantly, no correlation of age at presentation with lung function parameters at T_0_ was evident: *r* = 0.149, 0.103 and 0.042 for FVC%, FEV_1_% and FEF_25/75_%, respectively.

Longitudinal models, revealed: significant non-linear effects of age at first presentation on the average change of FEV_1_% and FVC% between T_1_ and T_0_ (*p* = 0.0001 and 0.004, respectively); evidence of interaction effects between age at presentation and the value at baseline, for FEV_1_% and FVC% (*p* = 0.0002 and 0.030, respectively); a linear effect of baseline values emerged (*p* < 0.0001) for FEF_25/75_% only.

Notably, neither the baseline value or age at spirometry showed a significant effect on the average change between T_1_ and T_2_ for each lung function parameter (all p-values > 0.05). Therefore, the stability of lung function was based on the therapeutic management and tailored follow up.

For what concerns the effect of baseline value, children with lower FVC%, FEV_1_% e FEF_25/75_% at baseline have higher estimated average increases from T_0_ to T_1_ with respect to children with higher values at baseline.

Estimates of the effect of gender, BMI z-score and age at diagnosis are reported in Table 4.

According to the multivariable model, for FEV_1_% the effect of gender was associated to an estimated coefficient of 3.28. This means that, children with equal age at spirometry and baseline FEV_1_% average increase of FEV_1_% between T_0_ and T_1_ will be 3.28 points higher for the female than the male.

Greater increases in female vs male were estimated also for FEF_25 − 75_% but not for FVC%.

In the same model the estimate of the effect of BMI z-score is 1.72. This means that, by comparing two children with the equal values of FEV_1_% at baseline and age, the first having 1 unit of BMI z- score higher than the second child, we expect an average increase of FEV_1_% of 1.72 points higher vs. the second child. Moreover, FVC% change increased according to BMI z-score increase.

**Table 4:** Effect of gender, BMI z-score, and age at diagnosis, on the change of lung function parameters (T_1_-T_0_)

Response variable	Covariate	Unadjusted models		Multivariable models	
		Est (95% CIs)	*p*-value	Est (95% CIs)	*p*-value
FVC %	^a^ **Gender** **BMI z-score** **Age**	0.60 (-2.52, 3.72) 1.87 (0.24, 3.50) 0.77 (-0.07, 1.62)	0.7041 0.0243* 0.0752	0.64 (-2.41, 3.69) 1.75 (0.14, 3.36) 0.68 (-0.81, 1.54)	0.6821 0.0333* 0.1219
FEV_1_%	^a^ **Gender** **BMI z-score** **Age**	3.34 (0.60, 6.07) 1.78 (0.41, 3.14) 0.12 (-0.71, 0.94)	0.017* 0.0107* 0.7824	3.28 (0.58, 5.98) 1.72 (0.36, 3.08) 0.09 (-0.75, 0.93)	0.0173* 0.013* 0.8339
FEF_25/75_%	^a^ **Gender** **BMI z-score** **Age**	5.28 (1.20, 9.36) -0.07 (-2.36,2.22) -1.03 (-2.37,0.32)	0.011* 0.950 0.140	5.12 (1.10, 9.14) 0.01 (-2.38, 2.41) -0.93 (-2.25,0.38)	0.013* 0.992 0.165

In the first column (labeled with ‘unadjusted models’) estimates and p-values were obtained by separate models, in which each of the above features was added to the pertinent longitudinal model, i.e., the model for the pertinent lung function parameter. In the second column, estimates and p-values were obtained by adding all the features in the pertinent longitudinal model

^a^gender: females VS males, * *p* < 0.05

### Relationship between lung function parameters and radiological score

A poor negative correlation resulted between the modified Reiff score and FVC%, FEV_1_% e FEF_25/75_% at baseline (Spearman coefficients: rho=-0.156, -0.204, -0.103, respectively). The distribution of the modified Reiff score may have affected this result given that in our case series the radiological score showed mild lung impairment.

## Discussion

BE are characterized by wet/productive cough along with recurrent exacerbations. Children/adolescents with BE require developmentally appropriate care, support and supervision from clinicians and their families. However, mild radiographic bronchial dilatation is reversible if treated optimally early [1, 8]. Few and sparing data are currently available on non-CF BE outcomes in children [1].

In particular, few heterogeneous evidences are reported on the preservation/deterioration of lung function in non-CF BE diagnosed and managed in early childhood. Most authors reported a median age at diagnosis of 7–9 years by HRCT [13–15, 35]. *Gaillard et al.* [8] considered a case series of 22 children, 50% early infants (< 4 years) who had at least two CT scans of the lungs over 6 years of clinical practice.

Only one study [43], retrieving data from three different countries (Alaska, Australia and New Zealand), considered a sub-cohort of 87 children (66% of overall case series including either subject with chronic suppurative lung disease, lacking of HCRT evidence of BE) diagnosed at early ages (range of median age when first HRCT scan confirmed BE, across the three countries: 2.1–5.7 years). In this study children experienced a common pathway of early and recurrent pneumonia requiring hospitalization with subsequent development bronchiectasis and chronic respiratory morbidity.

As well as, our study reported results of children with a diagnosis of non-CF BE confirmed by HRCT at early ages (median age 4 years) and followed for 2.5 years from diagnosis.

In this population the most frequent cause of non-CF BE is post-infectious, in agreement with previous studies [44–48], chronic cough and a history of recurrent bronchitis, pneumonia, and wheezing are the most frequent symptoms raising suspicion of non-CF BE.

The lung involvement at diagnosis described in our population resembles the results described in the literature [49–51]. BE are unilaterally localized, especially affecting the middle lobe (44.1%) and the left lower lobe (17.4%); the upper lobes are relatively spared. Only in a minority of patients (11.8%) the BE are bilaterally localized. Similar findings are reported in the cohort described by *Eralp et al.* [52], even if a significant percentage of that population was affected by PCD.

The lung function trajectory in non-CF BE children has not yet been thoroughly characterized. The study designs are heterogeneous and the results are rather conflicting [15, 17–20, 44, 50, 52, 53]. According to *Twiss et al.* [20], the parameters of FVC%, FEV_1_% and FEF_25/75_% were reported to drastically decline over time and the post-infectious etiology was the main determinant in lung function deterioration. Other studies showed that the lung function of patients with non-CF BE is generally normal at diagnosis and remains stable 3–5 years after diagnosis [16, 43, 50, 52, 53]. In particular, *Ullmann et al.* [44], reported that the FEV_1_% and FVC% parameters did not significantly change with respect to the baseline, in the case of post-infectious BE and PCD. A reduction of FEV_1_% and FVC% was detected in patients with secondary immunodeficiencies, monitored for 10 years after diagnosis.

Lung function monitoring in children allows to understand lung function trajectories and disease course which in turn are essential to optimize treatment strategies [7].

In our population, mainly free of comorbidities, with a median age at diagnosis of 4 years, characterized by a mild disease according to the modified Reiff score, we had evidenced that pulmonary function parameters improve more in patients having lower values of FVC%, FEV_1_% and FEF_25/75_% at baseline (T_0_). This was in agreement with data on lung function trajectories described *by Kapur et al.* [15], in children, with a median age at diagnosis of 8 years and followed for 5 years from diagnosis.

The improvement of lung function parameters was recorded 1 year from the spirometry at baseline, thereafter the trend was stable.

The evidence that a full recovery of pulmonary function is detectable within 1 year from baseline spirometry, could be explained by assuming that non-CF BE of post infectious etiology may not represent a progressive disease. Earlier and appropriate management likely improves the clinical outcome [1, 3, 12–14]. This should be further confirmed by the low exacerbation rate requiring hospitalization reported in our study (21%) and by its comparison with data reported in the literature.

Indeed, the early therapeutic approach with the antibiotic treatment with three-times-weekly oral azithromycin for six months may have influenced the clinical improvement reducing the number of exacerbations in the first two years of follow up [54, 55].

It is difficult to compare our exacerbation and re-hospitalization rates with other studies, due to the lower age of children at diagnosis. *Kapur et al.* [15] and *Ullmann et al.* [44], reported data on older children at diagnosis (7–9 years) and with age increase a major number of respiratory exacerbations of post-infectious BE is managed in the outpatient clinic [15, 44]. Indeed, the improvement of bronchial hyperreactivity, the maturation of the immune system, and the increase of the airways lumen with growth are all factors that contribute to reducing the frequency of respiratory exacerbations over time [44].

Anyway, our data may be compared to the one reported by the study of *Mc Callum et al.* [16], according to the short term follow up and young age at diagnosis: our data of 21% of re-hospitalization rate may be considered the result of an early and adequate management. Moreover, *Eralp et al.* [52] reported a significant reduction in exacerbations rate after diagnosis.

Our data suggest also a gender different response to treatment and lung function improvement that need to be confirmed by further studies. Genetic and epigenetic and/or hormonal factors involved in bronchiectasis disease susceptibility could explain this difference.

The radiological diagnosis of BE in children relies largely on adult criteria not validated in children and this may lead to over-diagnosis [8]. There are no radiological scores currently validated for the pediatric ages [56]. However, we have resorted to the modified Reiff score since recently reported to be a good tool not only for the diagnosis of BE, to quantify lung involvement from a radiological point of view, but also the degree of pulmonary function impairment [21–24, 26].

The relationship between lung function and radiological scores has been previously explored, but conflicting results have been published [53]. Some authors [48, 55, 56] report a significant relationship between the radiological severity of BE and the reduction of pulmonary function parameters, while others have found only a slight association [57–60]. This latter was confirmed also by our results.

Noteworthy, spirometry parameters detected at baselines, in our case series were not associated to hospitalization, likely due to the use of a more aggressive therapeutic approach in the worse cases at diagnosis.

The main strengths of this investigations are: (1) the evaluation of children diagnosed at early ages, (2) the robust statistical evaluation of the evolution of lung function parameters as outcome to define the appropriate management of BE, and (3) the evidence of a mild relationship between lung function at first spirometry and radiological score defined at diagnosis.

Our data pointed out the importance of an early diagnosis and management as recommended by recent ERS guidelines, considering the evidences of reversibility of the bronchiectasis, especially in younger children and those with lesser radiographic severity [61]. Children were evaluated by expert staff and the diagnostic investigations and the therapeutic approaches are those reported in the current guidelines [1, 4, 5].

However, the study was retrospectively conducted in a single tertiary care center; therefore, our findings need to be confirmed by further prospective, multicenter studies in order to obtain data from different population to make generalizable these preliminary findings. A multicenter national cohort would be also able to provide useful information about socioeconomical risk factors and possible inequalities in our country [62].

The main limitations are the lack of microbiological data, the retrospective study design and the length of follow- up. To critically appraise the first limitation, we have to consider that all children were submitted to laboratory/clinical tests to exclude any etiology different from the post-infective one. Indeed, at younger ages diagnostic bronchoscopy with bronchoalveolar lavage may be encouraged after a careful consideration of which patient needs this procedure and a rigorous estimate of its pros and cons [63].

We lost some patients during he follow up, probably due to the improvement of the disease course. Thus, we collected retrospectively the data available in that cohort. Therefore, our proposal will be to prospectively evaluate the respiratory function of patients with non-CF BE who are still undergoing follow up.

In our study children were followed up average for 2.5 years from the diagnosis (T_2_). This might be a limitation, but our data confirm that in a cohort with mild BE, the spirometry parameters improve within one year from baseline, and possible exacerbations requiring or not hospitalization fall within this time-window. The lung function parameters didn’t not further change and no further exacerbation has been recorded. Further prospective studies are warranted to confirm these findings.

## Conclusions

A stable pulmonary function is detectable within 2 years follow up from baseline spirometry, supporting the hypothesis that an early diagnosis and appropriate management improves the clinical outcome in post-infectious non-CF BE.

Moreover, our findings suggest that the modified Reiff score could be considered as a good tool not only to quantify the radiological lung involvement but also to estimate the degree of pulmonary function impairment through the correlation with the lung function parameters.

## Data Availability

The datasets used and/or analysed during the current study are available from the corresponding author on reasonable request.

## Abbreviations

non-CF BE: Non-Cystic Fibrosis Bronchiectasis
HRCT: High Resolution Computed Tomography
BMI: Body Mass Index
FVC%: Forced Vital Capacity
FEV_1_%: Forced Expiratory Volume in 1s
FEF_25/75_%: Forced Expiratory Flow 25–75% of forced vital capacity
MRI: Magnetic Resonance Imaging
PCD: Primary Ciliary Dyskinesia
BAR: Broncho-Arterial Ratio
WHO: World Health Organization
ATS: American Thoracic Society
ERS: European Respiratory Society
GINA: Global Initiative for Asthma
ANOVA: Analysis of Variance
GEE: Generalized Estimating Equations method
BPD: Bronchopulmonary Dysplasia
